# Degradable Polymeric Bio(nano)materials and Their Biomedical Applications: A Comprehensive Overview and Recent Updates

**DOI:** 10.3390/polym16020206

**Published:** 2024-01-10

**Authors:** Ketan Kuperkar, Leonard Ionut Atanase, Anita Bahadur, Ioana Cristina Crivei, Pratap Bahadur

**Affiliations:** 1 Department of Chemistry, Sardar Vallabhbhai National Institute of Technology (SVNIT), Ichchhanath, Piplod, Surat 395007, Gujarat, India; kck@chem.svnit.ac.in; 2 Faculty of Medical Dentistry, “Apollonia” University of Iasi, 700511 Iasi, Romania; 3 Academy of Romanian Scientists, 050045 Bucharest, Romania; 4 Department of Zoology, Sir PT Sarvajanik College of Science, Surat 395001, Gujarat, India; anita26p@gmail.com; 5 Department of Public Health, Faculty of Veterinary Medicine, “Ion Ionescu de la Brad” University of Life Sciences, 700449 Iasi, Romania; i.crivei@uaiasi.ro; 6 Department of Chemistry, Veer Narmad South Gujarat University (VNSGU), Udhana-Magdalla Road, Surat 395007, Gujarat, India; pbahadur2002@yahoo.com

**Keywords:** degradable polymers, chemical modification, polymeric nanoparticles, biomedical applications

## Abstract

Degradable polymers (both biomacromolecules and several synthetic polymers) for biomedical applications have been promising very much in the recent past due to their low cost, biocompatibility, flexibility, and minimal side effects. Here, we present an overview with updated information on natural and synthetic degradable polymers where a brief account on different polysaccharides, proteins, and synthetic polymers viz. polyesters/polyamino acids/polyanhydrides/polyphosphazenes/polyurethanes relevant to biomedical applications has been provided. The various approaches for the transformation of these polymers by physical/chemical means viz. cross-linking, as polyblends, nanocomposites/hybrid composites, interpenetrating complexes, interpolymer/polyion complexes, functionalization, polymer conjugates, and block and graft copolymers, are described. The degradation mechanism, drug loading profiles, and toxicological aspects of polymeric nanoparticles formed are also defined. Biomedical applications of these degradable polymer-based biomaterials in and as wound dressing/healing, biosensors, drug delivery systems, tissue engineering, and regenerative medicine, etc., are highlighted. In addition, the use of such nano systems to solve current drug delivery problems is briefly reviewed.

## 1. Introduction

A biomaterial is a natural or synthetic substance engineered to interact with biological systems for direct medical treatment. To qualify a substance as a biomaterial, it needs to be biocompatible and should be able to meet its desired function. Also, the physical, chemical, and biological properties of a substance to be used as biomaterial for tissue engineering and drug delivery devices affect the host response and thus need fine-tuning. Polymers and their composites provide an assembly of biomaterials with different physical, chemical, and mechanical properties. The degradable polymers undergo disintegration into low molecular weight products which are reabsorbed or excreted by themselves [1,2,3,4,5,6].

In the case of biodegradable polymers, the changes in the material properties take place by degradation over time in the presence of living organisms. Such changes may cause long-term host responses which are remarkably different from the initial response. Synthetic polymers are often non-degradables that create problems in waste disposal. However, to circumvent these issues, intense efforts have been made to produce degradable polymers. These polymers undergo thermal/photo/oxidative/hydrolytic/enzymatic biodegradation at random places along the macromolecular chain or through unzipping harmless products [7,8,9,10,11].

**Figure 1:**
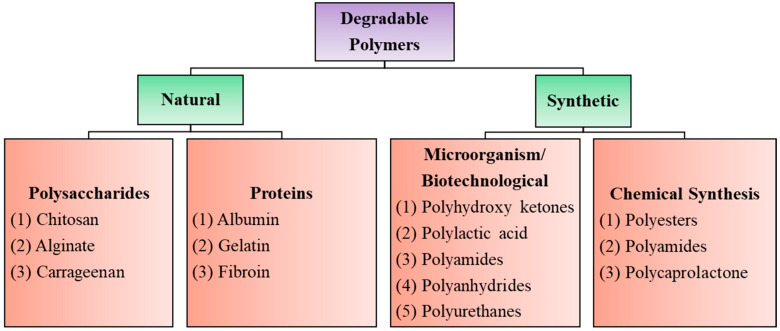
Degradable polymers of biomedical interest with a few well-known examples.

Biodegradable polymers are those that occur in nature such as polysaccharides, proteins, nucleic acids (in plants/animals), those synthesized by bacteria such as polyhydroxyalkanoates, or some semisynthetic materials derived from biopolymers or purely synthetic ones such as polylactic acid, polycaprolactones, etc., (Figure 1). Synthetic degradable polymers often belong to the polyester family, such as polyglycolides and polylactides. However, such polymers are poorly biocompatible, releasing acidic degradation products. Their poor processability and loss of mechanical properties during the initial stages of degradation also limit their usefulness as biomaterials [12,13,14].

**Figure 2:**
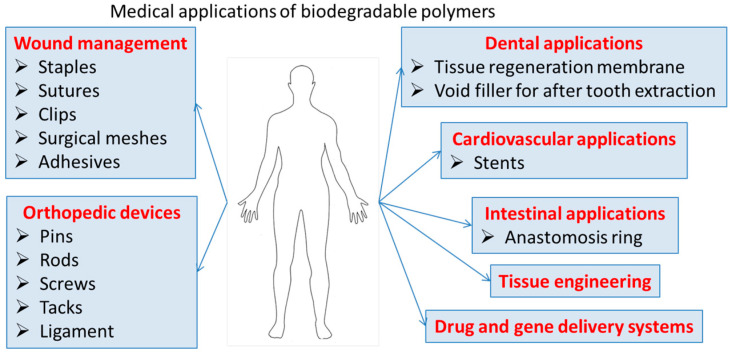
Medical application of biodegradable polymers.

The biocompatibility, low toxicity, and chemical functionalities of these polymers can lead to nanomaterials for a variety of biomedical applications, that ensure clearance from the body and eliminate the need for retrieval or explant. Degradable polymers contain polymer chains that are hydrolytically or enzymatically cleaved, resulting in soluble degradation by-products. These biodegradable polymeric materials with enhanced properties such as bioavailability, stability, and controlled release of bioactive compounds are highly useful in tissue engineering/cartilage scaffolds, creation of skin, blood vessels, temporary prosthetic implants, etc., as illustrated in Figure 2.

The clinical applications of these types of materials were reviewed by Stoppel et al. [15]. The chemical, physical, and mechanical properties of the biodegradable polymers might be suitable for their study in different types of medical applications. However, there are not enough data concerning the in vivo tests, especially for the degradation rate, in order to increase their translation to serious clinical assays. In fact, a systematic research strategy related to the modulation of their biodegradability in the presence of highly reactive oxygen species can be useful to widen the application fields of these materials.

Furthermore, the physical, chemical, and biological properties of degradable polymers can be improved and finely tuned through different strategies such as blending with other substances to form polymer alloys or nanocomposites, physical/chemical cross-linking to produce hydrogels, forming interpenetrating networks (IPNs), using their functionalities, and making useful block and graft copolymers as well as self-assembled nanoaggregates. It is therefore not surprising that concrete efforts have been made in recent times to generate such useful macromolecular biomaterials. The thermal and mechanical properties, degradation, and surface characteristics can be optimized on their nanoconstructs using chemical modification strategies. Physicochemical cross-linking, polyion complexes, IPNs, blends/nanocomposites with other materials, coating on to some nanoparticles, and layer-by-layer (l-b-l) assembly may lead to multiphase polymer systems with desired properties for their biomedical applications [12,16,17,18,19,20,21].

Drug delivery systems (DDS) require information on high drug uploading, programmed target-specificity, cellular uptake, clearance, toxicity, metabolism, pharmacokinetics, and excretion. An ideal delivery system should increase the efficiency of the drug and facilitate controlled release from a biocompatible nanocarrier to increase patient compliance. An important factor contributing to DDS efficacy is the passive accumulation of these particles at the desired site through the enhanced permeability and retention (EPR) effect. The conventional DDS have immediate release and sometimes are toxic as the frequency of their administration must be high in order to reach the therapeutic level. In order to avoid these drawbacks and to increase the pharmacokinetics, such as blood circulation, biodistribution, and active targeting, second and third generations of DDS were investigated [22]. The surfaces of these particles were modified in order to improve their stealth effect and also to increase their targeting delivery. A stealth effect can be induced by using PEG as a hydrophilic block as this polymer limits the plasma protein adsorption by steric repulsions and rapid clearance by the reticuloendothelial system (RES). This effect avoids the recognition of the particles by the immune system and thus their blood circulation is enhanced, allowing their accumulation in highly vascularized areas. Besides the particle’s surface, the stealth effect can be also influenced by the shape, size, and core composition [23]. However, until now, the number of formulations based on biodegradable polymers translated to clinical assays is quite limited.

Several nanomaterials from organic (polymers, amphiphiles) and inorganic (nanocarbon, silica, and metal/metal oxide nanoparticles) compounds have been examined as carriers for DDS. These include soft core–shell nanoaggregates such as liposomes, polymer micelles, and polymersomes (resulting from self-assembly of amphiphilic block or graft copolymers), nanoparticles, nanocapsules, nanogels, nanofibers, dendrimers, and nanocomposites which have been of immense use as nanocarriers for drug delivery systems. However, the size and polydispersity/shape/surface charge/surface characteristics/ porosity, etc., need to be fine-tuned for its desired application purpose. Several review articles had already been published on the use of different types of materials for the preparation and characterization of DDS [24,25,26,27,28,29,30,31,32,33,34].

Advances in polymerization techniques in the past two decades have led to the synthesis of tailor-made and well-defined polymers/copolymers of predetermined molecular weight and composition. Several biodegradable, stimuli-responsive, amphiphilic, and double hydrophilic copolymers; and polysaccharide-based graft copolymers, polymer bioconjugates coupled with modern characterization techniques, particularly self-assembled nanoaggregates; have been rather promising in recent years to boost drug nanocarrier research. Biocompatible polymers, due to their characteristic features, have been of immense interest in the scientific community for the diagnosis and treatment of several diseases. This article provides an updated comprehensive overview on all aspects along with discussion on the recent literature studies relevant to degradable polymers in this context.

## 2. Degradable Polymers

**Figure 3:**
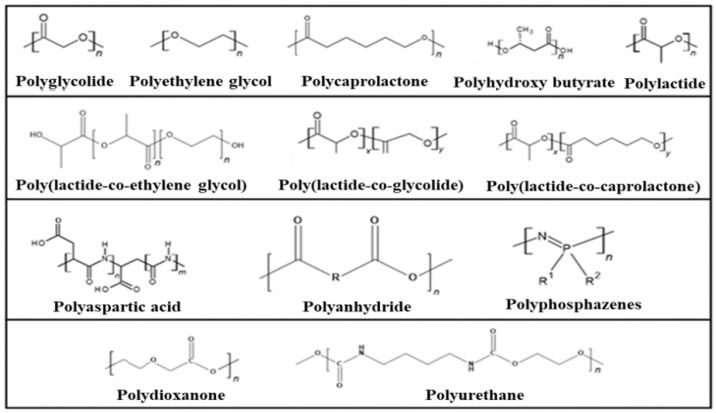
Synthetic degradable polymers of biomedical interest.

The most important examples of synthetic degradable polymers of biomedical interest are provided in Figure 3.

### 2.1. Bacterial/Synthetic Degradable Polymers

#### 2.1.1. Polyesters

Polyester-based biodegradable polymers have been useful as medical devices such as sutures, plates, stents, screws, bone fixation devices, and tissue repairs, due to their desirable physical and physicochemical properties. A few well-known polyesters are polylactic acid (PLA), poly(lactic-co-glycolic acid) (PLGA), poly(ε-caprolactone) (PCL), poly-3-hydroxybutyrate (or poly-β-hydroxybutyric acid (PHB), poly(3-hydroxybutyrate-co-3-hydroxyvalerate) (PHBV), poly(propylene carbonate) (PPC), poly(butylene succinate) (PBS), and poly(propylene fumarate) (PPF).

Polyhydroxyalkanoates (PHA) polyesters are produced by microorganisms via fermentation. These polymers had been approved by the United States Foods and Drugs Administration (FDA) for clinical applications and used in orthopedics, repair patches, tissue engineering applications, sustained and controlled drug delivery, biomedical devices, and artificial organs [35]. Several bacterial (both gram-positive and gram-negative) species can produce PHA. The degradation of PHA proceeds via an abiotic route through hydrolysis of ester bonds. These novel materials find applications in orthopedics, tissue engineering, drug delivery, biomedical devices, and artificial organs [36,37,38,39,40,41,42,43,44,45,46].

**Figure 4:**
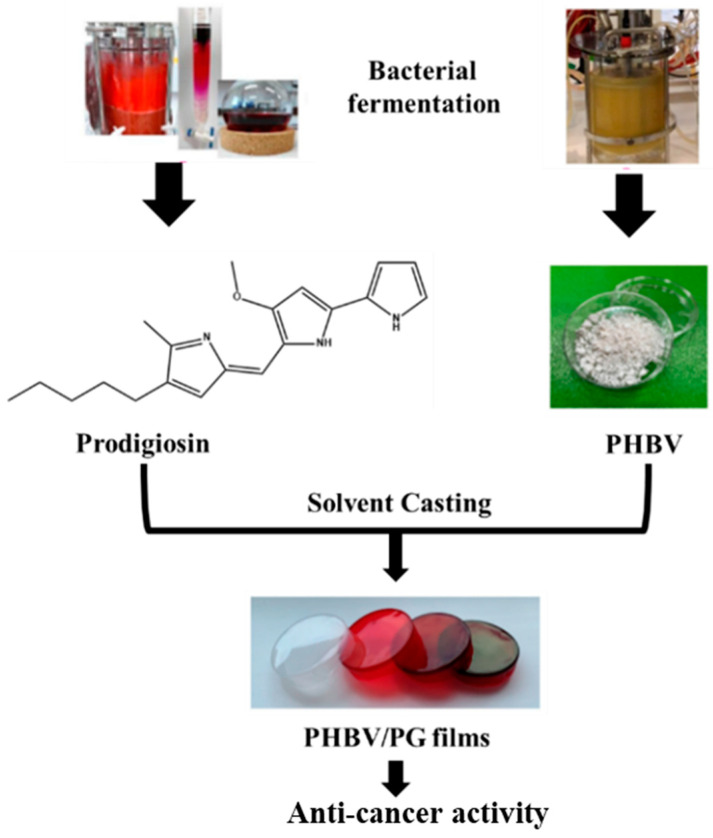
Study design and potential biomedical application of the PHBV films with incorporated prodigiosin (PG).

In a very recent study, a bacterial polyester PHBV and bacterial pigment Prodigiosin (PG) were produced using a simple solvent casting method [46], as illustrated in Figure 4.

Polylactic acid (PLA), polyglycolic acid (PGA), and polylactic acid-co-glycolic acid (PLGA) are FDA-approved polyesters. PLA is a naturally occurring aliphatic polyester obtained from wheat, rice bran, potato starch, corn, and biomass. PLA’s monomer is 2-hydroxypropionic acid, a chiral molecule existing in D- and L- and DL form, and therefore its properties depend on stereoregularity in the polymeric chain. Its biocompatibility, low cost, and biodegradability make it a widely explored biomaterial for various biomedical applications such as therapeutics, surgical implants, prosthetic devices, tissue culture, resorbable surgical sutures, drug delivery systems, etc. [47,48,49,50,51,52,53,54,55,56].

PGA is highly hydrophilic and can be produced by ring-opening polymerization of glycoside (a cyclic diester of GA) or by polycondensation. It is employed as a filler with other biodegradable polymers or in tissue scaffold application [57].

PLGA is a copolymer and is also important biomedically. Copolymerization by changing the monomer ratio of two monomers can result in a product with better mechanical and chemical properties without affecting biodegradability and biocompatibility.

Poly(ε-caprolactone) (PCL), a semi-crystalline degradable polymer, is of interest for long-term implantable/drug delivery systems as it is used in many FDA-approved surgical implants and drug delivery devices for tissue engineering and regenerative medicine applications. For example, the FDA has approved PCL for sutures with a trade name of Maxon™. It can be produced in high yield by ring-opening polymerization of ϵ-caprolactone. Being hydrophobic, PCL has a glass-transition temperature of −60 °C and melting point of 63 °C [58,59,60]. PCL copolymers have been good in accelerating the rate of biosorption. For example, copolymers of ϵ-caprolactone with dl-lactide are more flexible and have a higher degradation rate than PCL. It has been widely used in tissue engineering due to its availability, and the cheap and simple chemical modification approach by which physical/chemical/biological properties can be adjusted. By copolymerization with other esters, the biodegradability rate can also be adjusted [61,62]. Also, its high permeability to various agents has made it an important precursor for the development of drug delivery systems [63,64,65,66,67].

Polydioxanone (PDX) is a poly(ether-ester), has a glass-transition temperature in the range of 10 °C and 0 °C, and about 55% crystallinity. It is a commercially available biodegradable polymer that can be spun into fibers and is thus useful as monofilament suture, medical devices, in tissue engineering, and in drug delivery applications. These electrospun fibers are tough and possess mechanical properties similar to structural components of native vascular extracellular matrix (ECM). PDX sutures are non-antigenic and non-pyrogenic, possessing often greater pliability and strength than polypropylene and other sutures. Moreover, they retain their strength for longer periods than other synthetic absorbable sutures. The presence of an ether oxygen group in the polymer backbone renders PDX-based sutures with good flexibility. They have a unique shape-memory property and so have emerged as important biomaterials [68,69,70,71].

Polyphosphoesters are composed of phosphorus-incorporated monomers and consist of phosphates with two (one in the backbone and one in the side) groups. Polyphosphoesters are biocompatible like natural polymers. The hydrolytic cleavage of the phosphate bonds in the backbone occurs easily. These polymers have high potential for drug and gene delivery as well as scaffolds in bone tissue engineering [72,73,74,75].

Aliphatic Polyester Nanoparticles: The case of polylactic-acid-based nanoparticles.

Nanoparticles (NPs) from degradable polymers are promising as drug delivery carriers for controlled/sustained release due to their improved pharmacokinetics by maintaining the drug concentration at a therapeutic level with minimal side effects. The polyesters used as NPs for drug encapsulation/entrapment are FDA approved. Aliphatic polyesters contain several hydrolytically degradable ester linkages under physiological conditions; non-toxicity to human connective tissue degradation products make these nanoparticles biocompatible. The drug release profile resulting from the degradation of NPs depends on the crystallinity, glass-transition temperature, hydrophobicity, and molecular weight of the polymer and the mechanism can be explained using different models for predicting the kinetics of drug release [76,77,78].

Small sizes of NPs lead to a high surface area to volume ratio and thus enhance the absorption potential and cytotoxicity. However, aliphatic-polyester-based NPs may possibly lead to a reduction in cytotoxicity due to their high biocompatibility and biodegradability. These improve their bioavailability, drug payload and targeted delivery, and stability in the bloodstream.

NPs of aliphatic polyesters are commonly prepared via an emulsion process. Polymeric NPs refer to both nanospheres and nanocapsules. The drug molecules are uniformly distributed in the polymeric matrix of nanospheres, while the drug gets encapsulated inside the core cavity which is surrounded by a polymeric membrane that acts as a thin shell.

The preparation of such nano templates depends upon the hydrophilic/hydrophobic nature of the drug as well as the polymer solubility. Polyesters are hydrophobic and therefore drug solubility is often correlated to the formulation of nanoparticle type. Water-soluble hydrophilic drugs may produce nanocapsules while the hydrophobic ones will form nanospheres. The preparation of NPs is simple. Basically, here both the drug and polyester are mixed to uniformly distribute the drug in the polymeric matrix. The main challenges are the control of particle size and efficient drug loading. The commonly used methods to obtain NPs are briefly described here. In a single-emulsion/solvent-extraction method, usually polyester and the hydrophobic drug are dissolved in a water-immiscible organic solvent and emulsified with water containing surfactant. The organic phase is removed through evaporation at low pressure or vacuum, or by solvent extraction. Nanoparticles are recovered by centrifugation and washed with water or buffer solutions to remove any traces of solvent, stabilizer, and free drug prior to lyophilization. However, for hydrophilic drugs, the method is not suitable. Another approach involves double emulsions which also offer nanoparticles loaded with both hydrophobic and hydrophilic drugs. In this method, an aqueous solution of the hydrophilic drug is added to water immiscible organic phase containing the dissolved polymer and the w/o emulsion formed is subsequently added to a second water phase containing an emulsifier which, on mixing, forms a w/o/w emulsion. A similar solvent removal and purification process is then employed. These methods are quite simple but several process parameters such as phase volumes (oil and water), polymer, drug and stabilizer concentration, type of solvents, and stirring rate need to be carefully controlled. Other methods based on nanoprecipitation, salting out effect, dialysis, spray drying, and supercritical fluids are also employed in the preparation of polyester NPs. Supercritical-fluids-based methods are good due to the use of environmentally friendly solvent and providing NPs with no traces of residual solvents. NPs are stabilized by adsorbed amphiphilic molecules on their surface as the encapsulated drugs need to be protected from degradation. The flocculation of dispersed NPs may produce macroscopic lumps that lead to the early diffusion of drug molecules and degradation. The low molecular weight ionic surfactants can lead to high zeta potential to the dispersed particles and stabilize them through electrical repulsion. Amphiphilic block copolymers (BCPs) anchor to the particle surface and result in steric stabilization. However, care needs to be taken of colloid stability by adsorbed polymers as, if not carefully chosen, the adsorbed polymer may exert the opposite effect, causing flocculation by bridging mechanism or through depletion flocculation. Adsorbed polyelectrolytes (natural or synthetic), as well as block/graft copolymers with a polyelectrolyte block, may render stability to electrosteric stabilization. The stability of dispersed systems has been extensively examined quantitatively after the DLVO theory in the 1940s.

Polylactic-acid-based NPs have been extensively studied in the context of their biomedical application as these NPs have shown a great potential as nano-reservoirs for hydrophobic drugs, proteins, and genes, and releasing the active compound at the targeted site at the desired rate (thanks to their interesting properties). This led to a growing interest also in the nanomedicine field for the synthesis of nanoparticles for drug delivery and imaging purposes [79,80,81,82,83,84].

Block and graft copolymers of polylactic acid with a hydrophilic polymeric moiety behave like amphiphiles and thus self-assemble in an aqueous solution to form polymer micelles or polymersomes. These core–shell aggregates from amphiphilic polymers are excellent for solubilization of hydrophobic drugs [85,86,87]. In particular, PLA copolymers, such as those with polyglycolic acid and polyethylene glycol, have been very interesting as these can improve the characteristic properties of nanoparticles and fine-tune their applications. This co-amphiphilic entity may be water soluble if the lactic acid mole ratio is low. The form nanoaggregates and the hydrophobic drug is solubilized in the polylactic acid core that is stabilized by covalently bonded PEG shell [88,89,90,91,92]. The chemical nature adjustment by the proper ratio of comonomer results in interesting physical/chemical/biological properties in the NPs which would be desirable for their biomedical applications. Several studies have reported the NPs of polylactic acid-polyglycolic acid copolymers using varying ratios of lactic acid and glycolic acid to achieve improved results [93,94,95,96,97,98,99,100,101,102]. PLA graft copolymers also form multiphase polymer systems with an enormous possibility of improving the desirable features of polylactic acid in biomedical applications. The PLA-based graft copolymers are categorized on the basis of different monomers/polymers attached to PLA. That can be prepared using different grafting strategies. These PLA-based graft copolymers with biomacromolecules such as chitosan, cellulose, dextran, starch, etc., as well as synthetic polymers like polyethylene glycol, and vinyl-based polymers have been reported in the literature [103,104,105,106,107,108,109].

Polylactic-acid-based nanocomposites with other organic or inorganic nanomaterials have been important as these help improve the properties of PLA and make PLA-based nanocomposites popular in biomedical fields including bone substitute and repair, tissue engineering, and drug delivery system. Nanocomposites with PLA or PLA copolymers as matrixes significantly help improve PLA’s properties and therefore studies have been made to produce useful nanocomposites with clay minerals, carbon nanotubes, and graphene, etc. [110,111,112,113].

#### 2.1.2. Polyamino Acids

Polymers based on amino acid monomers are derived from the polycondensation of amino acids and have been widely investigated in drug delivery applications due to their low toxicity, extreme biocompatibility, and biodegradability that make them attractive biomaterials, and so are employed in medicinal, biomedical, pharmaceutical, and cosmetic applications [114,115,116,117,118].

In particular, the natural polyamino acids such as poly(β-alanine), poly(lysine), poly(γ-glutamic acid), polyaspartic acid, polyhistidine, and polyarginine; and synthetic polyamino acids such as poly(hydroxyethyl-l-asparagine) and poly(hydroxyethyl-l-glutamine) are explored widely for different applications. These polymers offer multiple pendant functional groups for drug attachment, resulting in polymeric prodrugs. For example, polyglutamic acid-texane has been developed for the first-line treatment of non-small cell lung cancer. Poly(l-glutamic acid) has already entered into Phase III clinical trials [119,120]. Natural amide-based polymers such as gelatin and albumin are widely used in the preparation of biodegradable microcapsules and microspheres.

Synthetic poly(amino acid)s are also investigated in biomedical/drug delivery applications as they are structurally similar with naturally occurring proteins. In general, the application of water-soluble polymer conjugates in drug delivery via carrier-based systems offers several advantages such as improved drug pharmacokinetics, minimal toxicity to vital organs, tissues, or cells, and enhanced drug build-ups at the target site. Moreover, the release of the drug from a carrier at the target tissues can be programmed to achieve a predetermined drug delivery.

Low molecular weight drugs are typically delivered using poly(amino acids), which are cleaved by enzymes into non-toxic substances. Poly(amino acid) polymers degrade inside the body, resulting in the amino acids that are biologically active and are often used as drugs. The following section highlights a few widely used poly(amino acid) polymers in drug delivery.

Poly(aspartic acid) is widely used in biomedical applications, especially gene delivery and drug delivery, due to its low toxicity profile, non-antigenic, excellent biocompatibility, and biodegradability. Poly(aspartic acid)s like other poly(amino acid)s are extensively used in several areas such as water treatment, paper processing, and paint additives. In the field of biomedical sciences, poly(aspartic acid) is employed in the design of dialysis membranes, drug delivery systems, hydrogels, orthopedic implants, and artificial skin [121].

#### 2.1.3. Polyanhydrides

Polyanhydrides can be aliphatic, aromatic, unsaturated, hybrid, or blend depending on the desirable physical properties suiting for specific applications. Polyanhydrides used as biomaterials are surface-eroding polymers that contain two carbonyl groups bound together by an ether bond. Polyanhydrides have been used for the delivery of bioactive molecules to various organs (brain, bones, blood vessels, and eyes) of the human body and are often fabricated as micro/nanoparticles to allow for injectable, oral, or aerosol delivery due to superior biocompatibility and lack of toxicity [122].

These are degradable polymer materials that possess hydrolytically labile chemical bonds in their backbone and can be broken down without secondary influence. They easily undergo hydrolytic degradation to their respective diacids, which are completely eliminated from the body within a short period of time. The degradation of the anhydride bond varies with polymer backbone chemistry which is important for their drug delivery applications. It has been found that these biocompatible polymers degrade into non-mutagenic and non-cytotoxic products [123,124].

Polyanhydride-based NPs can be successfully used as drug carriers. Betulin and its derivatives (e.g., betulin disuccinate) have a broad spectrum of biological relevance, including anti-cancer activity, thus these compounds are promising as new, potentially therapeutic agents. The major problem, which limits their potential pharmaceutical uses, is facing the poor aqueous solubility of lupane triterpenes when trying to formulate pharmaceutical compounds from betulin. However, this problem can be solved by obtaining a polymeric form of betulin and forming it into nanospheres, thus nanoparticles prepared from betulin-based polyanhydrides may have significant applications in drug delivery systems [125].

#### 2.1.4. Polyphosphazenes (PPZ)

Polyphosphazenes are inorganic polymers comprising a backbone of repeating phosphorus and nitrogen atoms with alternating single and double bonds. The two electrons of phosphorus can be used for side-chain conjugation, while two electrons of nitrogen remain a lone pair. By incorporating different side groups, or by using two or more co-substituents in the PPZ backbone, derivatized PPZs offer a wide range of properties. These can be hydrophobic/hydrophilic/amphiphilic, crystalline/amorphous, and bioinert/bioactive. Changes in the organic side groups allow substantial alteration in the physical and degradation properties of these polymers which can be rendered as useful and versatile biomaterials with desired mechanical properties and degradability for tissue engineering applications, such as bone tissue or blood vessels in bioimaging, phototherapy, dentistry (restorative composites and dental liners), and medical devices. PPZs incorporated drugs into their structure by physical encapsulation or chemical bonding, form a prodrug delivery system. The possibility of synthesizing materials with tailored degradation kinetics and their structural flexibility makes PPZs an attractive choice as an efficient carrier in drug delivery. However, these polymers are expensive and are used only for limited purposes [126,127,128,129,130].

Compared to other synthetic polymers, such as polylactic acid (PLA) and poly(lactide-co-glycolide) PLGA, the advantages of PPZ are remarkable, due to the controlled tuning of physicochemical properties via the side-chain conjugation. Furthermore, PPZs are hydrolytic and biodegradable materials yielding non-toxic degradation products. Also, a range of PPZ-based biomaterials has been researched for the development of scaffolding materials for use in bone and skeletal regenerative tissue engineering [131,132,133].

#### 2.1.5. Polyurethanes

Polyurethanes are obtained from the reaction of diisocyanates with compounds containing active hydrogen atoms such as diols or diamines. Polyurethanes have been of great interest due to their versatility, biocompatibility, and tunable mechanical properties and porosities. The major applications include flexible and rigid foams, adhesives, and surface coatings. These have long been used in the biomedical field as a thermoplastic elastomeric material for cardiovascular applications. Catheters, heart valves, prostheses, and vascular grafts have all typically been made of biostable and inert polyurethanes. Being a very stable material, there has also been a rise in research interest in recent years in building resorbable/degradable polyurethanes for tissue engineering and drug delivery systems [134,135,136]. It is possible to create biomedical polyurethanes by giving their backbones hydrolysable segments (e.g., polyether, polyester, and polyamide segments). The use of biocompatible aliphatic or cycloaliphatic diisocyanates (ICs) and diisocyanates generated from amino acids is a second method for producing such polyurethanes. Some substances, such as toluene 2,4-diisocyanate and 4,4′-methylene bis(phenyl isocyanate), are less hazardous than conventional ICs. Such polyurethanes have also been shown to increase cell adhesion and proliferation while having no negative side effects.

Depending on their component parts, biomedical polyurethanes can be biodegradable or non-biodegradable polymers with biocompatible properties that can be adjusted to biological systems, such as those of the blood, organs, and tissues. Also, it is very simple to change the mechanical properties of polyurethanes, i.e., materials for implants with the best yield modulus, strength, and resistance to fatigue, wear, or friction can be accounted where the modulus, mechanical strength, and fatigue resistance are very essential and crucial parameters for polyurethanes to be used in reconstructive surgery of soft tissue and cardiovascular surgery. Due to these advantages, polyurethane-based nanoparticles are widely utilized for controlled delivery of proteins, growth factors, antibiotics, antitumor drugs, and other bioactive substances [137,138,139,140].

### 2.2. Biopolymers

#### 2.2.1. Polysaccharides

Polysaccharides are natural polymers that have gained enormous interest in recent years due to their abundance and having several functional groups such as hydroxyl, carboxylic, and amino groups. These entities are inert, cheap, non-toxic, and biocompatible with good water stability. These can be easily derivatized, cross-linked, or transformed into multiphase polymer systems such as interpenetrating networks (IPNs), polyblends, graft, and block copolymers [141,142]. Charged polysaccharides form useful polyion complexes. Important polysaccharides of biomedical interest such as cellulose, lignin, starch, agarose, CD, guar-gum, hyaluronic acid, chitosan, alginate, carrageenan, fucoidans, chondroitin sulfate, and pullulan are included for discussion herein.

Cellulose

Cellulose is the most abundantly found polysaccharide in nature, with sequences of β-D-glucopyranose units linked by β-(1,4) glycosidic bonds, and is a very promising advanced polymeric material. It can undergo degradation in the presence of bacteria and fungi present in the air, soil, and water. The biocompatibility, low toxicity, and low cost make cellulose and its derivatives a versatile precursor. However, extensive intermolecular hydrogen bonding transforms cellulose into a highly crystalline polymeric material and thus it is insoluble in almost all solvents, leading to difficult processing. Such dissolution of cellulose is circumvented using ionic liquids and deep eutectic solvents. Cellulose can be etherified or esterified to yield useful environment-friendly materials like methylcellulose, hydroxypropyl cellulose, carboxymethylcellulose, cellulose acetate, cellulose nitrate, etc. Cellulose nanomaterials such as cellulose nanofibrils and nanocrystals as well as bacterial nanocellulose have been of great research interest. However, cellulose has limitations such as a lack of thermoplasticity, poor crease resistance, and a tendency to cause antigenicity [143,144,145,146].

Lignin

Lignin is the other most abundantly found biopolymer that constitutes approximately 15–35% of the lignocellulosic biomass. Its high carbon content, greater thermal stability, enhanced adhesive properties, biodegradability, biocompatibility, non-toxicity, cost effective production, and biological activities make lignin and composite materials important for biomedical applications in tissue engineering scaffolds, wound dressing materials, and drug delivery carriers [147,148,149,150].

Starch

Starch is a plant polysaccharide made up of linear chains of amylose (soluble starch) and branched amylopectin components from a-glucose unit and is chiefly found in cereal grains, legumes, roots, and fruits. Amylose has glucose units connected by α-(1,4) linkage while amylopectin contains glucose units by means of α-(1,4) and α-(1,6) bonds. Starch obtained from different plant sources has different properties which also depend on the degree of maturity of the plant. Chemically modified starch and its physical blends or IPNs with improved properties are important biomaterials in bone and tissue engineering. However, starch is brittle and degrades before its melting temperature. Its poor mechanical properties and difficult processability limit its use as a pure material [151,152,153,154].

Agarose

Agarose is an uncharged polysaccharide, is a main constituent of agar (generally extracted from cell walls of certain marine red algae), dissolves in hot water, and also in polar non-aqueous solvents and ionic liquids. Structurally, it is a linear neutral polysaccharide consisting of alternate (1,3)-β-d-galactopyranose and (1,4)-linked 3,6-anhydro-α-l-galactopyranose units. It dissolves in boiling water and forms gels on cooling to ~<40 °C. Agarose forms chains which create flexible fibers. The chains can curl to helix structures and form very strong gels which display a pronounced hysteresis. Agarose is non-toxic and is useful as a gelling agent as commonly used in nucleic acid electrophoresis, affinity chromatography/ion exchange chromatography, gel plates, or overlays for cells in tissue culture, cell culture media, immunodiffusion techniques, etc. It has been used as a bio-ink due to its excellent biocompatibility, mechanical strength, and thermo-reversible low gelling temperature of 32 °C (this depends on concentration). The gelation results in the formation of an infinite network of 3D agarose fibers. Such hydrogel networks disassemble above 85 °C [155,156,157,158].

Cyclodextrins

Cyclodextrins are oligosaccharides composed of glucose units (α-D-glucopyranose) connected by α-(1,4) bonds enzymatically obtained as α-cyclodextrin (6 glucose units), β-cyclodextrin (7 units), and γ-cyclodextrin (8 units). The unique truncated cone-like structure of cyclodextrins possess an internal non-polar hole with polar hydroxyl groups placed on the surface of its molecule, and thus hydrophobic substances like drugs can get encapsulated in its cavity due to hydrophobic interaction and bind through operative forces like the van der Waals interaction and dipole–dipole interaction, thus forming a host–guest supramolecular complex. The chemistry and applications of cyclodextrins in different fields have been reviewed and these inclusion complexes can be derivatized and suitably chemically modified [159,160].

Guar-gum

Guar-gum is a readily water-soluble, high molecular weight polysaccharide extracted from *Cyamopsis tetragonolobus* seeds of the leguminous plant. It is made up of a main chain of D-Mann pyranose residues connected by β-(1,4) glycosidic bonds, linked to D-galactopyranose residues by α-(1,6) glycosidic bonds. It is a good emulsifier, thickener, and stabilizer in the food, pharmaceutical, and cosmetic industries. Its solubility in cold water increases depending on the galactose/mannose molar ratio. It is functionalized (esterified/hydroxyalkylated/carboxymethylated) and derivatized using different strategies to decrease its aqueous solubility and improve its mechanical properties for its use in biomedical applications. Guar-gum and its derivatives are designed for oral drug delivery due to enhanced stability over a wide pH range [161,162,163,164,165,166,167].

Hyaluronic acid

Hyaluronan or hyaluronic acid is a linear, non-sulfated glycosaminoglycan composed of disaccharide repeat units of β-1,4-D-glucuronic acid and β-1,3-N-acetyl-D-glucosamine linked by ß-1,4-glycosidic bonds. Hyaluronic acid is the major component of the extracellular matrix of vertebrate soft connective tissues where it is present in high concentrations in synovial fluid, vitreous humor, skin, and umbilical cord. It is commercially extracted from rooster combs or through bacterial fermentation. It is an anionic polysaccharide which can absorb a large amount of water and hence acts as lubricant in native extracellular matrixes controlling the viscoelasticity of connective tissues. Hyaluronic acid can be chemically modified by cross-linking and grafting. Hyaluronic acid is a major ligand for CD44 (frequently over-expressed on the tumor cell surface) and CD168 which are over-expressed in various tumor and inflammation conditions. Hyaluronic acid interacts with cells in many biological processes such as tissue homeostasis, cell proliferation, angiogenesis, tumor invasion, matrix organization, and anti-apoptosis. Hence, it is used in skin rejuvenation, tissue engineering, molecular imaging, and drug delivery applications. Unfortunately, hyaluronic acid is susceptible to rapid degradation. The hydrogels based on hyaluronic acid are generally brittle, or easily dissolve in aqueous solutions. Derivatized hyaluronic acid and IPNs/PICs of hyaluronic acid are useful biomaterials, though high cost and poor mechanical properties are major crunches [168,169,170,171,172,173,174,175,176,177,178,179,180,181].

Chitin and Chitosan

Chitin, a major component of the shells of shrimp and other sea crustaceans, is the second most abundant biomacromolecule used in the food and agriculture, textile, and pharma industries. It is biodegradable, biocompatible, non-toxic, mucoadhesive, and can be easily extracted and chemically modified to yield a wide range of products useful as biomaterials. Chitosan, derived from crabs and cell walls of fungi, is produced commercially by deacetylation of chitin. However, the extent of deacetylation, the content of impurities, and the distribution of the molar mass of chitosan depend on the natural source of the primary material as well as the preparation method. It is a cationic linear copolymer polysaccharide made up of β (1→4) linked 2-amino-2-deoxy-D-glucose (D-glucosamine) and 2-acetamido-2-deoxy-D-glucose (N-acetyl-D-glucosamine) units by glycosidic bonds. The primary amino groups in the polymer backbone of chitosan provide a positive charge on its surface which facilitates the capability of forming inter and intramolecular H-bonding. It also possesses antimicrobial (fungi, bacteria, viruses) activity, making it useful in the biomedical field [182,183]. However, poor solubility at physiological pH is one of its major disadvantages [184,185,186,187,188,189,190,191,192,193,194,195]. Even if the synthesis procedure is not always reproducible, chitosan was tested for short periods on humans and no signs of allergic reactions appeared [196].

Alginate

Alginate is a water-soluble anionic polymer composed of α-L-guluronic acid (G) and β-D-mannuronic acid (M) residues linked by 1,4-glycosidic linkages. It is non-toxic, biocompatible, biodegradable, and can be produced at low cost from marine brown algae. Alginate is easily processed and has many different biomedical applications. Alginate-based three-dimensional scaffolding materials can be employed as hydrogels, microspheres, microcapsules, sponges, and foams for tissue engineering. Alginates, in combination with other polymers as chemically modified, are extensively used in food and biomedical processes. Physical or chemical modification can lead to derivatives having various structures, enhanced properties, and improved applications. For example, the mechanical strength, gelation property, and cell affinity can be finely tuned through combination with other biomaterials, immobilization of specific ligands such as peptide and sugar molecules, and physical or chemical cross-linking. However, susceptibility to hydrolysis in acidic environments, and fabrication due to its low solubility are challenging areas [197,198,199,200,201,202,203]. Different clinical investigations have been reported for alginate-based materials. For example, it was reported that sodium alginate in a dose of 60 mg/day decreased blood pressure after 2 weeks of treatment. Also, alginate-based foodstuffs NTCELL^®^ and DIABECELL^®^ are in an advanced stage of clinical investigation [204].

Carrageenans

Carrageenans constitute a family of linear sulfated polysaccharides that can be extracted from red seaweeds, also called Irish moss (belonging to red algae-Rhodophyta). These large and highly flexible molecules can curl to helical structures and thus form highly viscous solutions or viscoelastic/elastic gels. Carrageenans are composed of alternate units of β-d-galactose and 3,6-anhydro-α-d-galactose, linked by α-(1,3) and β-(1,4) glycosidic unions. Depending on the method and the algae from which the carrageenan is extracted, three main types of carrageenans can be obtained. For example, kappa (κ-1 sulfate group per disaccharide), iota (ι-2 sulfate groups per disaccharide), and lambda (λ-3 sulfate groups per disaccharide), although several other types are also reported. Carrageenans play an important role in plant growth promotion and are elicitors of resistance to pathogens and thus are crop protectants against a broad spectrum of plant pathogens. They also have excellent gelling, thickening, protein binding, and colloid stabilizing properties; and so are widely used in the dairy, food, and cosmetic industries; and also have applications in the pharmaceutical industry, as gel base, emulsion stabilization, etc. However, low gel strength, and anti-coagulant activity are its major drawbacks [205,206,207,208].

Fucoidans

Fucoidans refer to ionic polysaccharides which contain substantial percentages of L-fructose and sulfate ester groups, mainly derived from brown seaweed and some marine invertebrates (such as sea urchins and sea cucumbers). They are non-toxic, biodegradable, biocompatible, and FDA-approved compounds and so are used in many nutraceuticals and functional food products. However, the performance of Fucoidan depends on the species from which it is extracted. It can be transformed into nanoparticles and microparticles, films or hydrogels, and polyion complexes with cationic biopolymers, making it useful in pharmaceutical technology. Fucoidan-based particles serve as a vehicle for several drug and protein molecules. Additionally, fucoidans have been extensively studied due to their several interesting biological activities such as antioxidant nature, anti-inflammatory, anticoagulant, antithrombotic, antifungal, antivirus, antitumor and immunomodulatory against hepatopathy, uropathy and renal path, etc., that enable them as pharmaceutically important [209,210,211,212,213].

Chondroitin sulfate

Chondroitin sulfate is the major constituent of hyaline cartilage, located in cartilage and also at the calcification site of the bone. Structurally, it is a sulphated polysaccharide with repeating disaccharide units of β-1,4-linked-d-glucuronic acid and β-1,3-linked *N*-acetyl galactosamine and ß-1,4-linked-D-glucuronic acid with certain sulfated position(s). The two major chondroitin sulfates differ from one another in the position of the sulfates at position 4 or 6. The presence of polar carboxyl and hydroxyl functional groups facilitates the polymer to interact with other materials covalently/electrostatically. Its antioxidant, antiatherosclerosis, anticoagulant, antithrombosis, and negative immunogenic properties make chondroitin sulfate a useful biomaterial. It is a widely used material in the treatment of osteoarthritis. Chondroitin sulfate can target the CD44 receptors on cancer cells and be used for cancer therapy [214,215,216,217,218,219].

Pullulan

Pullulan is an α-glucose polymer with α 1→4, and a few α, 1→6 glycosidic bonds and is primarily produced from starch by the fungus *Aureobasidium pullulans*. It is an excellent material because it is biodegradable, biocompatible, and non-toxic, and it is produced from plant-based starch or sugars regenerated by natural photosynthesis of carbon dioxide and water. Pullulan is a water-soluble, odorless, tasteless, edible polysaccharide, and forms stable, viscous non-hygroscopic solutions. Its adhesiveness, non-immunogenic profile, non-mutagenic and non-carcinogenic qualities, and lower permeability of oxygen make it popularly used in food packaging, pharmaceutical, and cosmetics industries. Pullulan’s solubility and other characteristics can be tailor-made via chemical modification. Various derivatives of Pullulan can be conveniently prepared by cross-linking, esterification, or etherification, blending Pullulan with other polymers, forming IPNs and graft copolymers. This entity comes up with a few limitations; it is highly expensive and brittle with low mechanical strength [220,221,222,223].

Ulvans

Ulvans are complex sulphated polysaccharides obtained from the cell walls of the green algae of the Ulva genus which represent up to 50% of the dry mass. These are mainly composed of sulphated L-rhamnose of D-glucuronic acid and its C5-epimer L-iduronic acid (as found in animal glycosaminoglycans), and of a minor fraction of D-xylose. Ulvans display several physicochemical and biological properties that make these polysaccharides important for food applications [224,225].

Dextran

Dextran, a bacterial homopolysaccharide, is a generic term for glucans made by polymerization of α-d-glucopyranosyl moiety of sucrose catalyzed by the enzyme dextransucrase. It contains glucose units connected by α-(1,6) bonds in the main chain and by α-(1,4), α-(1,3), and α-(1,2) bonds in the branches. The degree of branching, molecular weight, and other properties of dextran changes with the microorganism employed. Dextran solutions have excellent rheological behavior and have larger plasma volume expanding efficacy. Chemical modifications to it involve the introduction of aldehyde, (meth)acrylate, thiol, phenol, and maleimide groups. Being a biocompatible and non-toxic polysaccharide, Dextran is widely used as an antithrombolytic agent, a bio-adhesive, in protein and drug delivery, in tissue-engineered scaffolds, in pharmaceutical, and biomedical applications. Dextran-based injectable hydrogels are also put forth as a site-specific, trackable, chemotherapeutic device. Additionally, Dextrans are known as blood plasma expanders. However, it suffers from disadvantages of high cost and non-availability [226,227,228,229].

Gum arabic

Gum arabic is a complex mixture of glycoproteins and polysaccharides (in particular arabinose and galactose). It is a water-soluble neutral polymer and has been used extensively as stabilizer, thickener, and emulsifier in food, cosmetics, and pharmaceutical industries. It has also been widely exploited as an effective excipient in accomplishing various nanoscale scaffolds for drug delivery and biomedical applications. Gum arabic can be cross-linked to hydrogels, blended with other polymers, used to form drug conjugates, and be attached to nanoparticles that may have biomedical applications [230,231,232,233,234,235,236,237,238].

Pectins

Pectins are polysaccharides present in the cell walls of higher plants. Their structures consist of D-galacturonic acid units connected by α-(1,4) bonds, forming a linear chain framework interrupted by highly branched regions. The composition of pectin differs according to its botanical source. However, a poor water-vapor barrier and low mechanical properties are its few limitations [239].

Gellan

Gellan is an extracellular anionic, linear heteropolysaccharide produced by Pseudomonas elodea. It contains two glucoses, one glucuronic acid, and one rhamnose molecules. A commercially formed deacetylated Gellan gum is a firm, thick, and brittle gel and is used in the food industry. This biocompatible polymer, being FDA approved, is used in additives in foods, cosmetics, pharmaceuticals, and drug delivery systems [240,241,242], and has been thought to be relatively expensive [243].

#### 2.2.2. Proteins

Albumins

Albumin is a protein present both in animal and plant physiological fluids/tissues and plays an important role including maintenance of osmotic pressure, neutralization of free radicals, binding, and transport of various substances like hormones, drugs, in blood, etc. Albumins act as an interface between cells to various scaffold materials as collagen, thereby mediating the integration of these two components in tissue engineering. Serum albumin is a non-toxic, stable, and biodegradable protein. Its binding with drugs plays an important role in pharmacokinetics and drug distribution/metabolism. As a consequence, albumins have emerged as a potential drug carrier and find numerous applications as theranostic agents, contrast agents, biosensors, and implants against cancer, diabetes, arthritis, and hepatitis C. The structural domains and functional groups in albumin allow the binding and capping of several inorganic nanoparticles that enhances its bioavailability, compatibility, and circulation times, with selective bioaccumulation and reduced toxicity [244,245].

Silk proteins

Insects like silkworms and spiders produce silk, the toughest protein fiber in nature. Those produced by silkworms are primarily fibroin and sericin and have excellent mechanical properties like high tensile strength, flexibility, and resistance to compression, biodegradability, and minimal immunogenicity, the properties which make them biomedically important. Polymerization of enzyme fibrinogen gives fibrin (a protein that is known for its biodegradability and biocompatibility) which is an excellent source for the regeneration of bone. Fibroin is a glycoprotein which is insoluble, non-toxic, less immunogenic, hydrophobic, and histocompatible. It is extracted from silkworm cocoons and is made up of amino acids (alanine, serine, and glycine) resulting in antiparallel beta-sheet formation. It forms hydrogels, membranes (films), gels, nanoparticles, scaffolds, and fibers. The mechanical strength, malleability, and high surface area coupled with its biodegradability and biocompatibility make fibroin a viable alternative for application in the field of drug delivery. Sericin, a hydrophilic protein, dissolves in water and functions as a glue. It exists in a partial unfolding state comprising a beta-sheet of 35% and random coil of 63%. As a nanoparticle, it offers inherent anti-tumor, and antioxidant activity. It is non-toxic to fibroblast cells [246,247,248,249,250,251,252,253,254,255,256,257,258,259,260,261,262,263,264].

Gelatin

Gelatin is derived from collagen via controlled alkaline, acid, or enzymatic hydrolysis. It has excellent biodegradability and biocompatibility and because of its availability at low cost, it is a versatile polymer to meet industrial demand. The mechanical properties, swelling behavior, thermal properties, etc., of gelatin depend upon the collagen source, extraction method, and degree of cross-linking. Gelatin has been used in the biomedical field as a matrix for implants, and as stabilizers in vaccines against measles, mumps, and rubella. Due to its water permeability and solubility and multifunctional properties, gelatin finds its use as a drug-delivery vehicle. It produces a thermo-reversible gel which makes it a very good precursor in targeted drug delivery. Gelatin is available as drug carrier systems in the form of microparticles, nanoparticles, fibers, and hydrogels [265,266,267,268,269].

Elastin

Elastin, a structural protein, is the main component of elastic fibers present in the extracellular matrix (ECM). It imparts structural support to numerous organs and tissues. It is responsible for allowing tissues in the body to “snap back” to their original shape after being stretched or contracted. Elastin-like polypeptides are stimulus-responsive biopolymers derived from human elastin. Their low immunogenicity and control over their structure can lead to unique architectures as nanoparticles, nanofibers, and nanocomposites, and they display bioactive agents for their wide applications in tissue engineering and drug delivery [270,271,272,273,274,275,276,277].

Collagen

Collagen is the protein found in the human body that offers physical support to tissues by residing within the intercellular space. Generally, collagen is a rod-shaped polymer approximately 300 nm long with a molecular weight of approximately 300 kDa. Collagen undergoes enzymatic degradation within the body to yield its corresponding amino acids. Collagen plays a crucial role in preserving the biological and structural integrity of the ECM.

Animal-derived and recombinant collagens are valuable biomaterials for their use in cosmetic surgery and tissue engineering. Its composite with hydroxyapatite and tricalcium phosphate has been FDA-approved as a biodegradable synthetic bone graft substitute. Collagen has been used for the delivery of low molecular weight drugs, proteins, genes, and plasmids [278,279,280,281,282,283].

## 3. Chemical Transformation/Modified Degradable Polymers

### 3.1. Cross-Linking

Cross-linking resembles network formation through some physical or chemical means and is often desirable to impart desired mechanical properties and viscoelastic or even elastic behavior in polymer solutions. The labile bonds are frequently introduced through physical cross-linking and can be broken under physiological conditions, either enzymatically or chemically. Chemical cross-linking of polysaccharide using covalent cross-linking agents is a highly versatile procedure as it improves mechanical stability and rheological behavior. However, covalent cross-linking agents have been found to affect the integrity of the original polymer and may add toxicity in the final product [284,285,286,287,288,289].

**Figure 5:**
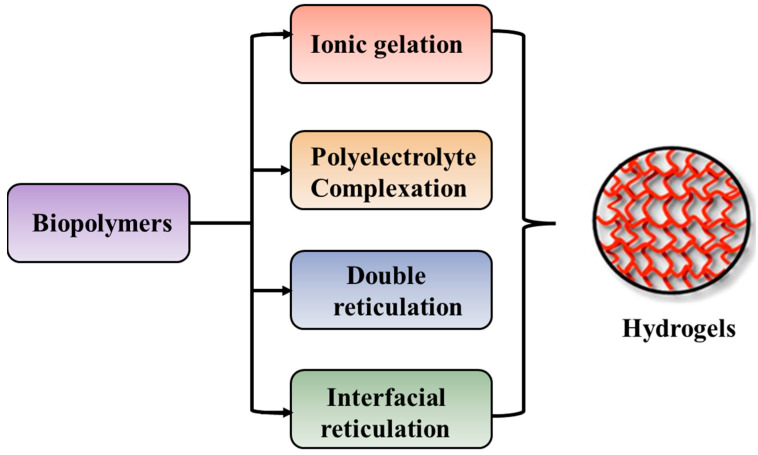
Preparation techniques specific for preparation of hydrogels starting from biopolymers.

The preparation of hydrogels, by cross-linking, starting from biopolymers such as polysaccharides and proteins, can be achieved by different methods as illustrated in Figure 5.

The ionic gelation method is represented by interactions between the positively or negatively charged polymers and cross-linking agents with complementary charges, whereas the polyelectrolyte complexation occurs by electrostatic interaction between oppositely charged polyions, in the absence of cross-linking agents [290,291]. Double reticulation or dual cross-linking is a novel method to apply two cross-linking factors (physical or chemical) to the same material. Generally, an ionic cross-linking is used first followed by a covalent cross-linking [292]. This method decreases the toxicity induced by the covalent cross-linking agents and increases the particle’s stability. The interfacial cross-linking method leads to the preparation of nanocapsules in the absence of any cross-linking agent [293].

Alginate can be easily cross-linked by ionic interactions in the presence of calcium ions at room temperature and physiological pH. Therefore, alginate gels are frequently used as matrixes for the encapsulation and release of bioactive molecules. Cationic polysaccharides, such as chitosan, are cross-linked by glycerol-phosphate disodium salt [294,295,296,297].

### 3.2. Polyblends and Biocomposites/Nanocomposites

Polyblends are physical mixtures of two or more polymers that are often used to improve performance properties of a material. Polymers can also be mixed with inorganic materials such as metal/metal oxide nanoparticles, silica, clays, etc., and form biocomposite/nanocomposite materials with superior and multifunctional properties. Biopolymers, for example, are beneficial for biomedical applications as they are not very rigid and often have poor mechanical strength as compared to synthetic polymers. In order to overcome these drawbacks, polyblend nanofibers prepared from the mixtures of natural and synthetic polymers represent biomimetic nanostructures that can substitute the native tissue, while providing the topographical and biochemical cues that promote healing. These have desired mechanical, structural, and biological properties suitable for tissue engineering and drug delivery systems.

Polymer-based biocomposites contain two or more different polymers bound together that exhibit superior and unique properties. Currently, a large variety of composites are being developed in order to design novel materials for biomedical and pharmaceutical applications. Nanostructured polymer composites that have superior performance and excellent mechanical properties, along with acceptable biological function, act as important biomaterials. Thus, polyblends and nanocomposites containing polysaccharides as one component, are of immense technological importance [298,299,300,301,302,303,304,305,306,307,308,309,310].

### 3.3. Interpenetrating Polymer Networks (IPNs)

**Figure 6:**
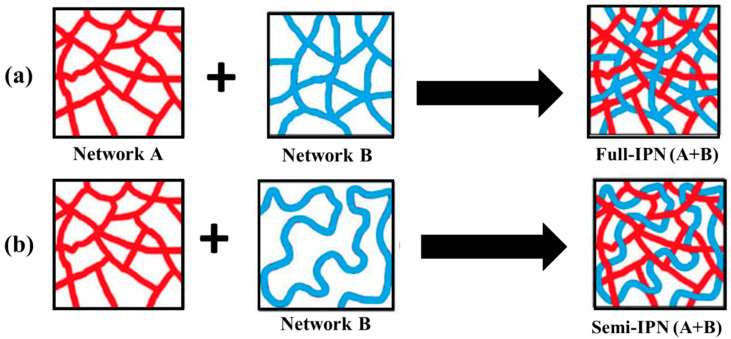
Schematics of the formation of (**a**) full interpenetrating polymer network (IPN) and (**b**) semi-IPN structures [311].

As illustrated in Figure 6, interpenetrating polymer networks (IPNs) are a blend of two or more incompatible polymers in a network with at least one of the systems synthesized in the presence of another. IPNs do form when an aqueous solution of a water-soluble polymer having a monomer is polymerized using an initiator. For example, an aqueous solution of polysaccharide in the presence of a vinyl/acryl monomer can be polymerized in aqueous media forming polymer chains that entangle with the polysaccharide chains [311].

An IPN is a multiphase polymer system that differs from polymer blend where an IPN swells but does not get dissolved in the solvents and, as a consequence, the flow/creep behavior is reduced. These IPNs are different from block and graft copolymers and even interpolymer complexes. In IPNs, only physical forces such as electrostatic, hydrogen bonding, etc., are involved which enable them to be used as a carrier system for drug delivery systems as well as scaffolds for tissue engineering. Also, IPN hydrogels have emerged as innovative biomaterials for drug delivery systems. In this context, polysaccharide-based IPNs are advantageous because of their remarkable properties like excellent swelling capacity, specificity, and mechanical strength. Chitosan and alginates are commonly used polysaccharides to produce IPNs [312,313,314,315,316,317,318,319,320,321,322,323,324,325,326,327,328,329,330,331].

### 3.4. Functionalization and Polymer Conjugates

**Figure 7:**
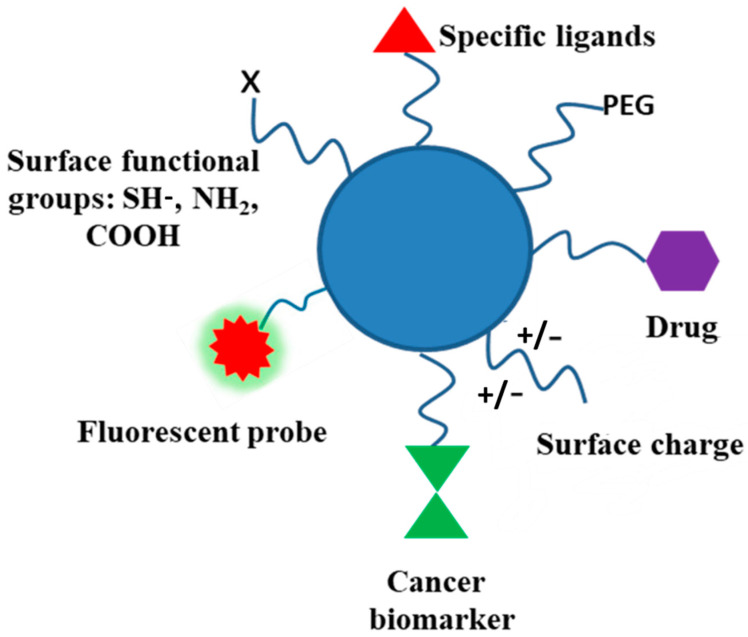
Schematic representation of functionalized NPs using different approaches.

Biodegradable polymers, and polysaccharides in particular, contain several functional groups and can be modified through covalent attachment of hydrophobic or hydrophilic substances. This renders interesting properties to them for biomedical applications. Chemical modification of polysaccharides (i.e., introducing hydrophobic, acidic, basic, or other functionality into polysaccharide molecules) can change their physical properties and solution behavior which can be employed for desired applications. Several strategies have been adopted for such chemical modification of polysaccharides. PEGylation, grafting of polymeric chains through polymerization, formation of polysaccharide-drug conjugates, etc., are the important examples of chemical modification of polysaccharides. The schematic representation of the surface functionalization of NPs is illustrated in Figure 7.

Several polysaccharides are water insoluble. Here, ionic liquids are used as appropriate solvents. Charged polysaccharides like chitosan or alginic acids can show marked solubility in water by altering the pH of the solution. Several inefficient methods have been developed for the modification of polysaccharides, as they produce waste products and may degrade the polysaccharide molecule. The presence of several hydroxyl functional groups along the polysaccharide backbone may use the saccharide oxygen as a nucleophile and may be etherified (with alcohols) or esterified (with acids, organic, or inorganic) at controlled degree/regioselectivity. Alkyl/benzyl/hydroxyalkyl/carboxyalkyl ethers, carboxylate/sulfonate, and other esters can be conveniently prepared. Saccharide carbon, as an electrophile, can replace polysaccharide oxygen by a heteroatomic nucleophile in a nucleophilic substitution reaction. These chemical reactions of polysaccharides may produce a variety of derivatives with better physical, chemical, and biological properties.

The introduction of reactive functional groups by alkylation and acylation, sulfation, phosphorylation, etc., can also have a profound effect on their intrinsic properties. The glycosidic bond can be destroyed by physical means such as ultrasound, photo, or microwave exposure, or can be hydrolyzed by the enzymes. The hydroxyl groups can be replaced by reactive azide, thiol, diol, and aldehyde groups. In the past decades, many enzyme-catalyzed modifications have been developed. Typical reactions include enzymatic oxidation, ester formation, amidation, glycosylation, and molecular weight reduction [5,10,12,332,333,334,335,336,337].

### 3.5. Polyelectrolytes (or Polyion) Complexes

Polyelectrolytes are water-soluble charged polymers. In aqueous solutions, they dissociate to form a macroion and counterion. The counter ion gets condensed on to the macroion or remains hydrated in the bulk solution. The solution behavior of polyelectrolytes is greatly influenced in the presence of salts and also by pH and temperature. Polyelectrolytes (both natural and synthetic) are highly useful in industries. Charged polymers can interact with oppositely charged species such as dyes, surfactants, drugs, and polymers, and form soluble polyion complexes or insoluble coacervates which are biologically important. For example, polyion complexes formed between oppositely charged polysaccharides such as chitosan and alginates, and those with charged proteins/polysaccharides with oppositely charged small molecules or polymers. Dilute solutions of a polyelectrolyte (e.g., charged polyelectrolyte or protein) when mixed with an oppositely charged low molecular weight or polymeric substance, led to a new phase which is formed by the spontaneous complexation through strong electrostatic interaction. However, a deeper understanding of the physical state (solid-like or liquid-like) is required. The effect of supporting electrolytes greatly influences the formation and properties of such complexes [338,339,340,341,342,343,344,345].

### 3.6. Block Copolymers

Biodegradable block copolymers have gained a medical and pharmaceutical research interest due to their tunable biocompatibility, biodegradability, and self-assembly properties. These polymers can be considered as suitable vehicles for drug delivery. The drug-loaded nanoparticles of these polymers get degraded in the biological environment and eliminated through the renal route. Block copolymers from degradable polymers are well-defined polymeric materials that can self-assemble to polymer micelles or polymersomes. These core–shell nanoscale structures are highly useful in biomedical applications. In particular, advances in polymerization techniques in the last two decades; such as controlled polymerization ATRP and RAFT processes, as well as the modern techniques available for characterization of their nanoaggregates; have led to an immense interest in biomedical application [345,346,347,348,349].

### 3.7. Graft Copolymers

Grafting offers a convenient approach to improve and increase the compatibility between synthetic and natural polymers. It is an effective and accessible method of chemical modification in polysaccharides. Several polysaccharides such as starch, chitosan, hyaluronic acid, cellulose, etc., have been used to achieve fruitful results of grafting. Hydrophilic or hydrophobic polymeric moiety can be grafted on the polysaccharide backbone by reactions with functional groups or polymerizing a monomer by creating active centers of polysaccharide chain via ‘grafting through’, ‘grafting on’, and ‘grafting from’ processes. Amongst these, the former approach is the most extensively studied. Grafting can be achieved by microwave or enzymatic synthesis. The microwave irradiation approach in grafting reduces the use of toxic solvents as well as cutting short the reaction time and increasing the grafting yield. Consequently, the microwave-grafted polymer exhibits superior properties as compared to polymer grafted by the conventional method.

In this way, polysaccharide-based graft copolymers show some interesting and improved properties such as water repellence, thermal stability, flame resistance, dye-ability, and resistance toward acid-base attack and abrasion. However, such behavior may be easily influenced by the presence of polar functional groups, high molecular weight, and relatively rigid backbone. The grafted copolymers possess many useful properties as compared to individual backbone or graft sequences. For example, grafting of a variety of vinyl monomers onto the backbone of different polysaccharides under different conditions of polymerization leads to the synthesis of new and interesting amphiphilic copolymers. However, it is difficult to precisely control and quantify the number and size of grafts, which hampers the reproducibility in the polymer. Hydrophobic grafts make the polysaccharide amphiphilic and thus can form core–shell polymer micelles. Grafting of synthetic polymers onto polysaccharides has been of interest to researchers because of their applications to the biomedical field as biomaterials or vehicles for drug delivery systems [350,351,352,353,354,355,356,357,358,359,360,361,362,363,364,365,366,367,368].

## 4. Mechanism of Polymer Degradation

Polymer degradation has often been regarded as a negative property but it has turned out to be quite beneficial in recent years particularly in connection with their biomedical applications. Often, it involves hydrolysis and oxidation and other physical/chemical/biological stresses. Hydrolysis refers to reactions with water that lead to cleavage of weak hydrolysable chemical bonds within a material. It has been studied extensively, especially for biodegradable polymers like polyorthoesters, polyanhydrides, polycarbonates, and polyamides. Hydrophilic polymers are very susceptible to hydrolysis and fast biodegradation of certain polymers occurs when the rate of diffusion and permeability are high. Also, the degradation process of polymeric products depends on the chemical structure and the degrading environmental conditions of polymers. The degradation is thus dependent on the polymer’s degree of crystallinity, cross-linking, porosity, mechanical stress, etc., and can be accelerated in the presence of a catalyst. The degradation leads to surface or bulk erosion involving the loss of material from the polymer bulk and changes in morphology, and crystallinity. Polymer biodegradation involves biodeterioration, bio fragmentation, and assimilation without neglecting the participation of abiotic factors. The knowledge of the degradation mechanism and kinetics of degradable polymers for biomedical applications are a prerequisite for their functioning.

Oxidative degradation of biomedical polymers such as polyurethane is yet to be fully understood. The oxidation mechanism involves biological defense action where the inflammatory cells generate oxidative agents that diffuse into polymeric implants and degrade them. Enzymatic degradation, which involves biodegradation of collagens, polysaccharides such as hyaluronic acids, some polyesters like polyhydroxyalkanoate, synthetic polycarbonates, and proteins, is also due to a defensive action. Several experimental studies on polymer degradation involving the mechanism, kinetics, and identification/analysis, as well as theoretical modeling, have been undertaken in the past and are not discussed here [369,370,371,372,373].

## 5. Preparation and Characterization of Polymeric Nanoparticles

**Figure 8:**
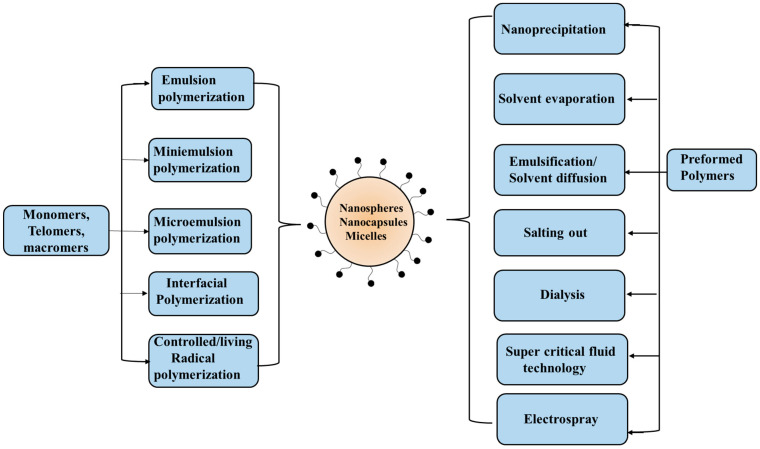
Preparation techniques specific for synthetic monomers and polymers.

Polymeric nanoparticles have become quite promising in recent years. Such entities made from biopolymers (proteins and polysaccharides) and synthetic polymers (including soft aggregates viz. polymer micelles and polymersomes from self-assembly of amphiphilic block/graft copolymers, and polyion complex micelles) are of great research interest. Fabrication of polymeric nanoparticles can be made possible by tuning some important parameters such as size, shape, and surface charge, along with high encapsulation efficiency for bioactive substances and their release profile. In fact, the size is very crucial. The nanoparticles from these biodegradable polymers and other materials based on them can be prepared using different procedures, as illustrated in Figure 8.

The most common preparation method of nanoparticles involves processes such as desolvation, coacervation, emulsification, and electrodynamic atomization. The desolvation process is a thermodynamically driven process that is induced by the addition of desolvating agents to produce nanoparticles. Here, the size is controlled by nucleation, condensation, and coagulation. The desired experimental conditions, such as amount of desolvating agent, solution pH, ionic strength, and nature/concentration of cross-linking agent, can be optimized for rapid nucleation and slow growth of the particle size that has control over the size/polydispersity. The emulsification phenomenon involves the preparation of liquid/liquid systems spontaneously formed using an aqueous and a non-aqueous (often non-toxic such as plant oils and surfactants) medium. The oil–water emulsion provides the stable nanoparticle dispersion with higher yield. Nanoparticles so prepared possess high loading and entrapment efficiencies. However, the use of toxic organic solvents and the large volume of water required are the drawbacks of this process. The coacervation approach is a phase separation technique that involves the two-phase separation of a molecularly dissolved polymer solution into a dense polymer-rich phase (coacervates), and another water-rich phase with low polymer content. Such coacervates can be produced by the addition of electrolytes (with salting out effect) and non-solvents, along with temperature changes (in case of thermo-responsive polymers). The coacervates are stabilized and further hardened by using chemical cross-linkers such as glutaraldehyde or formaldehyde, or by heat treatment. The mixing of aqueous solutions of two oppositely charged polymers (a polyanion like hyaluronic acid and a polycation like chitosan) also forms coacervates as a result of strong electrostatic interaction. The formation of soluble complexes or coacervates, however, depends on the relative amount of the two oppositely charged polymers. Electrohydrodynamic atomization or electrospray deposition involves the generation of a cloud of charge droplets using liquid atomization by high voltage electrical forces. Here, the nozzle tip sprays the charged particles, which further on during the process reach the substrate and produce nanoparticles. The particle size, charge, and droplet speed are regulated by varying the high voltage power supply. This approach is advantageous as it does not use any surfactant or other additives such as salt, oil, etc.

The information on the chemical composition, molecular structure, and purity of the polysaccharide is needed for cross-linking/blending with other polymers or inorganic nanoparticles, and more importantly, ligand attachment on chemical modification. Likewise, comprehensive characterization of such polymeric entities in terms of size/shape/charge, surface characteristics, and long-term stability/degradation on prolonged storage and rheological behavior is also necessary for their further evaluation for toxicity, drug loading, and targeting capacity. Various spectroscopic (FT-IR, Raman, NMR, UV/Vis, fluorescence) methods, in dispersed/dry medium, yield information on chemical composition and the functional group associated with the nanoparticles. The size/shape, polydispersity, and surface charge (as zeta potential) can be routinely measured by dynamic light scattering (DLS) (also called quasi-elastic light scattering (QELS) or photon correlation spectroscopy (PCS)). Here, this study provides the size/polydispersity of nanoparticles by measuring the diffusion coefficient and then using Stokes–Einstein equation. The monochromatic laser is used as a light source and the scattered beam is recorded effectively by two detectors mounted for direct (90°) or backscattered beams (173°). Besides the information on size and polydispersity from DLS, another important parameter in the characterization of the nanoparticle is the charge on its surface. It is often determined in terms of zeta potential. Several particle size analyzer instruments can be successfully employed to quickly determine zeta potential. Usually, a high zeta potential value on colloidal particles results the higher its dispersion stability. The presence of additives, like salts, and pH affect the zeta potential value.

Small-angle X-ray (SAXS) and neutron scattering (SANS) can be complementary techniques that provide accurate information on shape/core–shell morphology in self-assembled systems. Scanning electron microscopy (SEM) is a high resolution imaging technique based on a focused beam of a high energy electron beam. The secondary electron emitted during this interaction provides information on the geometry, topography, and composition of nanomaterials. Here, the image corresponding to the intensity of secondary electrons emitted at each interface is developed on the screen of a cathode ray tube. SEM is disadvantageous as the specimen or sample preparation is very tedious and requires extreme vacuum conditions and pre-sonication to avoid agglomeration. Likewise, Transmission electron microscopy (TEM) is based on interaction with a high energy electron beam similar to SEM. Here, a transmitted beam of electrons provides the final image. Here, the sample preparation is long and complex due to preparation of ultra-thin sections (≤100 nm) for smooth electron transmittance. Further advances in EM techniques like high resolution transmission electron microscopy (HR-TEM), cryo-EM for direct imaging, and tomography are becoming of great interest. Atomic force microscopy (AFM) is a non-destructive and non-invasive technique for biological and fragile polymeric specimens. It requires minimum sample preparation efforts with no necessary immobilization and even the non-conducting materials can be easily visualized without any prior surface treatment. AFM can be operated mainly in three modes, i.e., contact mode, non-contact mode, and tapping mode, each with useful information. It offers evidence to surface texture and roughness by providing high resolution 3D topographic images of the nanoparticles [374,375,376,377,378,379,380,381,382,383,384,385,386,387].

## 6. Drug Loading Efficiency and Release Profile

Drug loading and encapsulation efficiencies (DLE and DEE, respectively) are important parameters used for the characterization of a drug delivery system, and can be determined using the following equations:

$$DLE\%=\frac{amount\;of\;drug\;in\;the\;particles}{amount\;of\;added\;polymer+drug}\times 100$$

$$DEE\%=\frac{amount\;of\;drug\;in\;particles}{amount\;of\;added\;drug}\times 100$$

The encapsulation efficiency of drugs within the polymeric nanoparticles, or solubilized in self-assembled nanoaggregates, and the drug release profile depend on several factors. These have to be optimized in order to obtain a controlled delivery of drugs to the target site and desired doses. Other parameters, like size/shape/charge onto the nanoparticle, surface- or stimuli-responsive groups, and polymer biodegradation kinetics, also play an important role. Several experimental studies, as well as theoretical modeling in search of an appropriate design of nanoparticles, have been carried out. Precise control of drug release has a broad potential in the field of clinical medicine that can be of great help in drug delivery systems. There cannot be a single material or designed particles for different applications and thus each individual system has to be modified independently.

An ideal nanoparticle-based delivery system should have a high loading capacity. This would reduce the amount of the carrier required for administration. Drug loading is achieved by incorporating the drug at the time of nanoparticle preparation or by diffusion of the drug after incubating nanoparticles in the drug solution. However, the amount of bound drug and its interaction with nanoparticles depends on the chemical structure of the drug as well as the polymer. The study of the adsorption isotherms can detect the type of binding and the binding rate. The unbound drug is separated by ultracentrifugation or gel filtration, and from the amount of drug bound, the drug encapsulation efficiency can be calculated.

**Figure 9:**
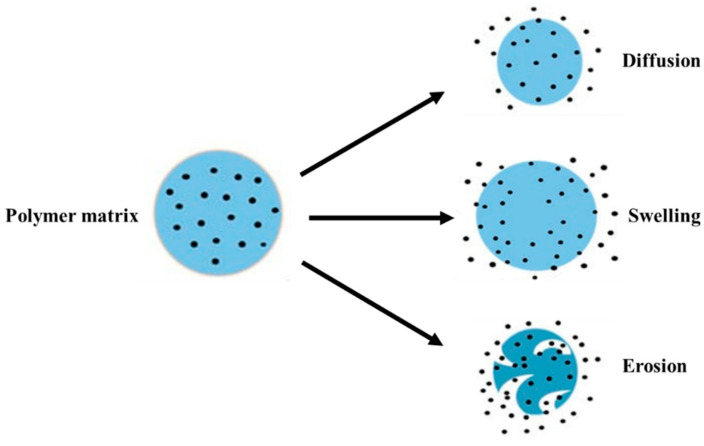
Schematic representation of the possible mechanisms of drug release from a polymer matrix.

For the drug release mechanism, the rate and timing of the drug release from nanoparticles is required. The mechanisms of drug release can be made possible following one or more methods viz. desorption of drug bound to the nanoparticle, diffusion through the nanoparticle matrix or the polymer wall of nanocapsules, or by the erosion of the nanoparticle matrix [388]. The schematic representation is provided in Figure 9.

The transport of a molecule by diffusion is related to a concentration gradient. In order to achieve the diffusion of a drug molecule through a polymeric matrix, the drug must be in a mobile (dissolved) state. Diffusion can occur with or without the swelling of the polymeric matrix. The swelling occurs when the polymeric matrix increases its volume due to the absorption of water. Hydrogels have the highest swelling capacity. The presence of water can activate the release of a water-soluble drug but also can control the release kinetics. The drug release kinetics are also controlled by the hydrolytic or enzymatic degradation of the polymeric matrix. This erosion can start from the surface, the bulk, or a combination of both.

The kinetics of the drug release depend upon the solubility and diffusion/biodegradation of the matrix materials, and the loading efficiency of the drug. In the case of DDS based on biodegradable polymers, drug diffusion, swelling, erosion, or dissolution of the polymeric matrix can occur simultaneously and may lead to zero-order release kinetics. The size of nanoparticles also affects the release; large size particles have a shorter initial burst release than smaller particles [389].

## 7. Toxicological Concerns

As such, degradable polymers bear no toxicological problems, but their chemically modified materials and nanocomposites may be risky for biomedical applications. A critical examination associated with these problems is thus necessary before putting them into tissue engineering/regenerative medicine, drug delivery vehicles, and other biomedical applications.

Nanomaterials, from inorganic, organic, or hybrid materials have been of interest in drug/gene delivery, tissue engineering, and bioimaging. However, the risks associated are adverse effects on interaction with living cells/organs. It is therefore necessary to examine their toxic effect and the health risks associated with these materials. Though nanoparticles based on biodegradable polymers may be regarded safe, the procedure used in their fabrication involving certain chemical processes may render them with some toxicity-related problems. For example, chitosan has been extensively used in the biomedical field although it has been shown to cause membrane damage. PGA has low solubility and a high degradation rate, with formation of an acidic product that can also provoke an inflammatory reaction. PLGA exhibits minimal systemic toxicity. Synthetic degradable polymers also may generate toxicity during their synthesis. Further, the products of hydrolysis of biodegradable polymers (carboxylic acid and/or hydroxyl chain end) may be oxidized, producing short chain carboxylic acid that may change the pH, which will further trigger an inflammatory response [390,391].

## 8. Biomedical Applications

**Figure 10:**
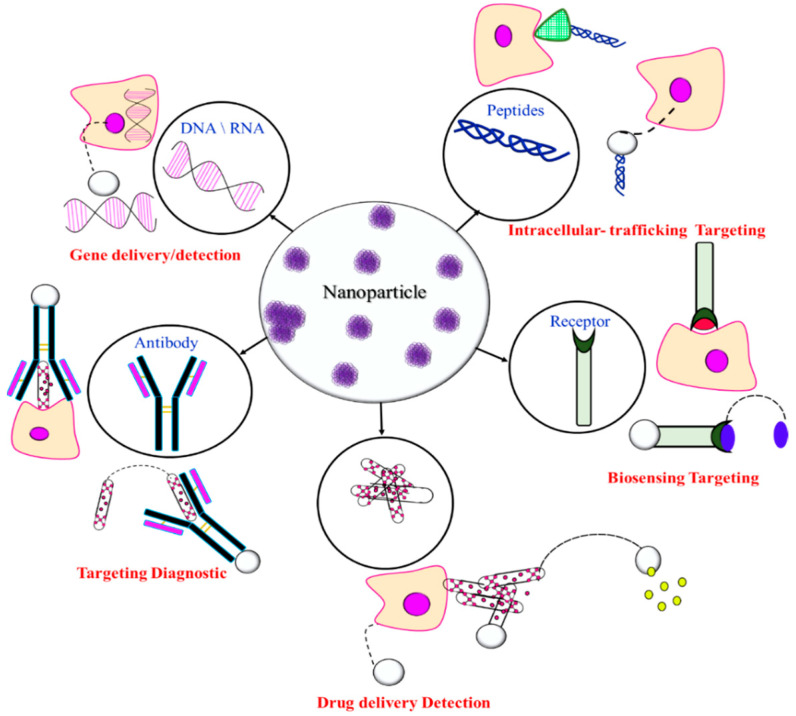
Degradable polymer nanoparticles of biomedical interest.

Uses of biodegradable (carbohydrate- and protein-based) nanoparticles are economically, socially, and environmentally sustainable and so are applied in wide areas. Also, a lot of research is ongoing in this direction. Their biocompatibility, biodegradability, and reduced immune response induction make them potential candidates for tissue engineering and in drug/gene delivery systems (Figure 10).

Some typical examples of studies investigating the use of biodegradable polymers in different biomedical applications are succinctly provided in Table 1.

### 8.1. Wound Dressing/Healing

Wound healing is a complex biochemical and cellular process that involves hemostasis, inflammatory, proliferative, and remodeling stages due to the physical disruption of tissue. Skin injuries constitute a gateway for pathogenic bacteria which produce virulence factors that impair tissue integrity. These pathogenic infections are usually associated with biofilm formation, where microorganisms interact to produce extracellular substances. A wide range of dressings are used to treat such severe wounds. The ideal dressing should have high absorption capacity, be comfortable, allow the visualization of the wound, and avoid pain in the removal, and should not provoke allergic reaction.

Polymers (both synthetic and natural) are used for the development of wound dressing foams, hydrogels, hydrocolloids, films, and membranes. Among these, polysaccharides such as alginate, cellulose, chitosan, and hyaluronic acid are versatile polymers for the development of formulations against skin infections. The efficacy of these biomacromolecules is related to their enhanced biocompatibility, biodegradability, non-toxic nature, chelation, presence of multifunctional groups, and ease of chemical modification [421,422,423,424,425,426,427,428,429,430,431,432,433].

### 8.2. Biosensors

Many diagnostic testing tools today include automated analyzers whose maintenance are very costly and time demanding. So, faster, smaller, and cheaper devices are much needed for laboratory testing. Nowadays, many medical conditions can be monitored by biosensors. A biosensor is a diagnostic device that detects specific biochemicals generated by the reaction between targeted analytes and the immobilized biomolecules, such as enzymes, antibodies, or receptors on the electrode. Biosensors show high specificity, fast response time, user convenience, and portability. They generally consist of biological elements that react with the target analytes and a transducer that transforms the signal that results from the reaction between the biomolecules to measurable or quantifiable values. They can be used for analysis of clinical samples or for ex vivo and in vivo monitoring of different physiological changes within the human body. Chitosan-based films have been applied to the cholesterol biosensor to improve its sensitivity [434,435,436,437,438].

### 8.3. Drug Delivery

The conventional and most convenient route of drug administration is through oral consumption. However, hydrophobic drugs show poor bioavailability, and those protein-based drugs are susceptible to enzymic degradation in the gastrointestinal tract. Hence, they have to be formulated carefully and must be administered via other routes such as infusions, injections, and implants. On administration of drugs though mouth or injection, the concentration is too high (resulting in toxicity) or too low for the drug to be effective. Thus, in order to circumvent this problem and maintain optimal drug concentration, novel drug delivery systems have been designed. These systems help in the release of bioactive substances at the precise rate and at the exact site while maintaining the optimum level of drug, leading to maximum efficacy and minimum associated side effects. In such controlled/targeted drug release formulations, one needs to encapsulate/solubilize the drug. Usually, nanosized materials such as solid lipid dispersions, liposomes, polymer micelles, polymersomes, dendrimers, inorganic nanoparticles, and mesoporous materials have been of great interest.

In this context, amphiphilic block (and graft, in particular), polymers are important. Their self-assembly to different size/shape nanostructures show high drug loading capacity and are versatile materials for the development of drug delivery systems. These nanoaggregates can optimize the drug release, protect it from degradation, and release rate/site-improving pharmacokinetics by prolonging drug circulation and capability of tissue targeting. Furthermore, the polymer micelles can be functionalized/cross-linked and used as mixed micelles and polyion complex micelles through chemical modification. Also, one or more blocks can be responsive to some stimuli, or biodegradable, and these additional features make these smart and intelligent materials good candidates for drug delivery systems.

Often thermo- and pH-responsive polymers are employed in targeted drug delivery systems. For example, the pH of cancerous cells is more acidic than the normal/healthier cells, and they also possess a higher concentration of glutathione (GSH) triphosphate than extracellular fluids, which are responsible for high redox potential in cytosol and nuclei. Degradable polymers have been promising materials for the design and development of new drug delivery systems. Due to degradation into biocompatible by-products, the biodegradable polymers formulated with drug constituents can be incorporated and used as drug delivery vehicles for the sustained release at the targeted site in desired concentration ranges within a therapeutic window [349,439,440,441,442,443,444,445,446,447]. In perspective, the main challenge is related to the preparation of multiple-functionalized DDS which will assure the on-demand drug release in a controllable manner under the influence of different types of external stimuli. The application of this type of smart DDS was very well reviewed in different papers [448,449,450,451].

### 8.4. Tissue Engineering and Regenerative Medicine

Tissue engineering is an interdisciplinary field that applies the principles of engineering and the life sciences to develop biological tissue substitutes that restore, maintain, or improve function. The new tissue is either grown inside a patient (in vivo) or outside a patient (in vitro) and then transplanted. The engineered tissue can also be used for diagnosis. Scaffolds play a crucial role in tissue engineering and biodegradable polymers. Both synthetic and natural polymers are important as scaffolding materials. An ideal porous scaffold is biocompatible, mechanically strong, biodegrades at the desired rate without producing any toxic substance, has a surface morphology favorable to interact with cells, acts as a template for the tissue, and supports cell attachment, proliferation, and differentiation.

The extracellular matrix (ECM) consists of a variety of proteins and polysaccharides that are assembled into an organized network that provides structural support to cells. Thus, protein- and polysaccharide-based materials have the potential advantage of supporting cell adhesion and function. Proteins such as collagen, gelatin, and silk; and polysaccharide such as alginate, hyaluronic acid, and chitosan are commonly used for scaffolds. However, complex structural features of proteins and polysaccharides, as well as immunogenicity and pathogen transmission, have generated interest toward synthetic polymers.

These polymers can have well-defined structural features, better processing flexibility, and no immunological concerns. Potentially useful synthetic polymers for tissue engineering are polyesters, polyanhydrides, polyphosphazenes, and polyurethanes.

Bone is complex living connective tissue that provides a mechanical chassis to tissues, anchor to muscles, and acts as a calcium storehouse. It is made of about 60% calcium phosphate minerals, 30% collagen-based matrix, and 10% of water. The minerals are composed of a mixture of tricalcium phosphate and hydroxyapatite, and provide rigidity and tensile strength (Young’s modulus 5–20 MPa). Bone defects caused by trauma, fractures, and tumors do not regenerate due to problems associated with regular autogenic and allogenic bone grafting methods. Thus, bone tissue engineering becomes important in this context.

Ceramic materials, polymers, and metals have been of interest as scaffolds which provide temporary support for cells, and to encourage cell separation and proliferation toward the formation of the desired new tissue. An ideal scaffold degrades when the support is no longer required. Also, its biocompatibility and pore size allow small biomolecules to pass to host tissue. Several inorganic materials such as steel and titanium and non-degradable porous composites have been used to heal the damaged bone by providing temporary support. Ceramics possess good osteoconductive and bone-bonding properties, but are brittle and show poor processing. These are, however, better as implants instead of their metal counterparts due to no distortion in magnetic resonance imaging, and reduce the cost and risk to the patient. Several degradable polymers and their composite materials have shown excellent mechanical and cell adhesion properties that suit orthopedic applications, implants, or devices. Magnesium-based composite materials possess good mechanical strength and are ductile and so have been found very useful in orthopedic uses. Besides use in scaffolds, this material can also be used as bone cement which degrades with time and promotes bone growth. Acrylate polymers polyblends starch/cellulose acetate have been found to be good materials as bone cements. Similarly, hydroxyalkanoate composites with silicate and borate with acrylic acid matrixes show enhanced mechanical properties [452,453,454,455,456,457,458,459,460,461,462,463,464].

### 8.5. Gene Delivery

Gene therapy combats inherited/acquired diseases like various genetic and neurological disorders, cancer, cardiovascular diseases, diabetes mellitus, etc. It involves therapeutic gene molecules (DNA and RNA) that can alter defective genes, modify missing genes, and silence mutated genes. However, the fragile nature of therapeutic genes and extracellular or intracellular biological barriers limit its use and therefore newer systems are being developed. Various types of non-viral vectors like cationic polyelectrolytes that form complexes with nucleic acids, and lipid-based nano-assemblies, as well as inorganic nanoparticles have been employed for gene-delivery applications, including nano-size cationic polyelectrolyte-siRNA/plasmid DNA/polyplexes. They are formed as a result of the electrostatic interaction taken up by cells through endocytosis mechanisms and thereafter polyplexes degrade, releasing the payloads. Polyelectrolytes such as polyethylene imines, poly(2-N-(dimethylaminoethyl) methacrylate), and poly(L-lysine) have been frequently investigated as polyplexes for gene delivery. The toxicity and degradability of polyelectrolytes need to be carefully ascertained and the molecular weight/chemical structure of the polymer needs to be carefully designed. Double hydrophilic block copolymers with one polycationic block and another neutral block may form complexes with nucleic acids and then undergo self-assembly to core-complex micelles which possess enhanced stability. Protein and polysaccharides are suitable vectors for their non-toxicity and biocompatibility.

Proteins have a 3D folded structure and are a major component of many natural tissues, are biocompatible, and biodegradable. Protein-based vectors, such as gelatin and albumin, have been widely employed for gene delivery. Cationic polysaccharides like chitosan are of interest as nano vectors due to their particular biological functions, such as cell signaling and immune recognition. However, due to its low pKa values (ca 6.3), chitosan has very limited solubility and low complexation ability to therapeutic genes in a physiological condition. Attempts have been made to chemically modify chitosan through functionalization or grafting [465,466,467].

### 8.6. Targeting Diagnostics

**Figure 11:**
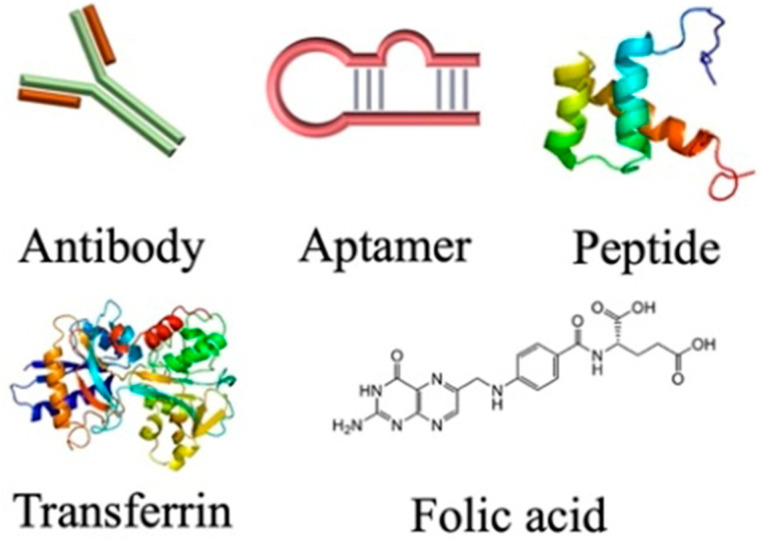
Types of specific ligands Redrawn from reference [468].

Targeted therapy is a type of treatment that uses nanoparticles functionalized with specific ligands in order to precisely identify particular receptors overexpressed in malignant cancer cells [405]. In order to prepare tumor-selective drug delivery systems, different types of ligands such as folic acid, peptides, transferrin, aptamers, and antibodies have been investigated, as illustrated in Figure 11.

Ligand-functionalized nanoparticles, obtained from biodegradable polymers, are very well suited for accomplishing anti-cancer targeting. The drug release from their nanoparticles directly to the tumor cells is facilitated by their high cellular uptake compared to non-functionalized particles. The immune cells are a critical target as the balance between an anti-cancer immune response and a tumor’s immunosuppressive microenvironment may show whether a tumor goes into remission or expands. Chitosan being a polybasic facilitates cellular uptake and adhesion to negatively charged mucosal surfaces, and is therefore well-suited for oral drug delivery [468,469,470,471,472,473,474,475].

### 8.7. Intracellular Trafficking

Cellular uptake and intracellular trafficking of nanocarriers are crucial in designing safe and efficient delivery systems. Intracellular trafficking can be optimized by tuning size/shape/charge and surface properties and is crucial for assessing their fate and toxicity [391,476,477,478].

## 9. Conclusions and Future Perspectives

Biodegradable polymers, both from natural and synthetic origin, and their derivatized products provide a plethora of materials with a great potential in biomedical applications. The cross-linking, interpenetrating polymer networks (IPNs), polyion complexes, polyblends, nanocomposites with organic and inorganic substances, and chemical modification to amphiphilic copolymers; and sometimes stimuli-responsive block and graft copolymers with interesting self-assembly; make these materials unique. These have been exploited in and as drug/protein delivery, gene transfection, bioimaging/diagnosis, tissue engineering, orthopedics biomedical devices, etc. The degradability, compatibility, non-toxicity, sterility, and stimuli-responsiveness show a wide scope of applications in the biomedical field. Interesting properties in these biodegradable polymers can be developed by physical or chemical modification such as cross-linking, making multiphase systems like polyblends and IPNs, and surface modification through functional groups, forming polyelectrolyte complexes through electrostatic interaction of charged biodegradable polymers with oppositely charged species producing amphiphilic or stimuli-responsive block and graft copolymers and their self-assembly in aqueous solutions. The present review focuses on the chemistry of degradable polymers and their derivatizing in different ways for potential biomedical applications such as and in devices, sensors, and tissue engineering with a focus on drug delivery applications. The advanced techniques in designing nanoparticles from degradable polymers and their chemical modification, coupled with their characterization for biomedical applications, inspires further improvements.

The use of these biodegradable polymers as DDS has immense potential, owing to their specific and superior features. However, there are some important challenges that must be overcome in the future. Until now, the majority of published studies focused only on the in vitro characterization of these systems. Therefore, at present, their practical applications and clinical translation are still quite scarce, with the exception of a few FDA-approved biodegradable polymers. Although there is clear progress in the synthesis of different types of DDS, their in vivo bioactivity, interaction with human cells, and long-term stability in a physiological medium should be investigated more profoundly in order to make them widely used in practical biomedical applications. Moreover, the multidisciplinary collaboration between polymer chemists, biologists, engineers, and physicians is indispensable for the synthesis of cost-effective and tailor-made DDS.

## Figures and Tables

**Table 1:** Recent examples of biomedical applications of biodegradable polymers.

Application	Polymer	Ref.
Wound dressing/healing	PCL/chitosan-carrageenan hydrogel	[392]
	Methyl cellulose	[393]
	Chitosan-PVA hydrogel	[394]
	Hydroxypropyl methylcellulose/carboxymethyl starch	[395]
	Sodium alginate-grafted poly(β-carboxyethyl acrylate-co-acrylamide) hydrogel	[396]
	Chitosan+hyaluronic acid hydrogels	[397]
Biosensors	Silk	[398,399]
	Nanocellulose	[400]
	Chitosan films	[401]
	Poly (vanillin-co-chitosan)	[402]
Drug delivery	Chitosan-based hydrogels	[403]
	Poly (HEMA-*co*-MAA) hydrogel	[404]
	PLLA	[405]
	Alginate	[406]
	Carboxymethyl cellulose-gum Arab-based hydrogel beads	[407]
Tissue engineering and regenerative medicine	PLA	[408]
	Methacrylated gelatin	[409]
	Chitosan	[410]
	Alginate/gelatine	[411]
	PLGA composites	[412]
Gene delivery	Hyaluronic acid-based microspheres	[413]
	Polylactic acid-b-poly(N-(3-aminopropyl) methacrylamide) copolymers	[414]
	Poly(amidoamine)	[415]
	Chitosan nanoparticles	[416]
Targeting therapy	Dextran	[417]
	Hyaluronan nanoparticles	[418]
	Carboxymethyl chitosan	[419]
	Polydopamine	[420]

## Data Availability

Not applicable.
